# A simple algorithm for computing positively weighted straight skeletons of monotone polygons^☆^

**DOI:** 10.1016/j.ipl.2014.09.021

**Published:** 2015-02

**Authors:** Therese Biedl, Martin Held, Stefan Huber, Dominik Kaaser, Peter Palfrader

**Affiliations:** a David R. Cheriton School of Computer Science, University of Waterloo, Waterloo, Ontario N2L 1A2, Canada; b Universität Salzburg, FB Computerwissenschaften, 5020 Salzburg, Austria; c Institute of Science and Technology Austria, 3400 Klosterneuburg, Austria

**Keywords:** Computational geometry, Weighted straight skeleton, Monotone polygon, Lower envelope

## Abstract

We study the characteristics of straight skeletons of monotone polygonal chains and use them to devise an algorithm for computing positively weighted straight skeletons of monotone polygons. Our algorithm runs in O(nlog⁡n) time and O(n) space, where *n* denotes the number of vertices of the polygon.

## Highlights

• We study the characteristics of the straight skeleton (SK) of monotone polygons.
• We devise an algorithm to compute the SK of monotone polygons in O(nlog⁡n) time.
• This algorithm can also compute the positively weighted SK in O(nlog⁡n) time.

## 1 Introduction

**Fig. 1:**
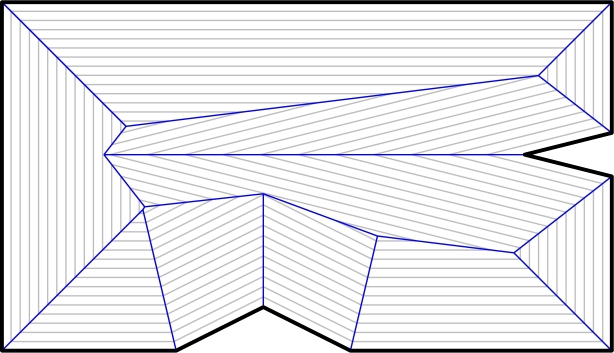
The straight skeleton S(P) of an input polygon P (bold) is the union of the traces of wavefront vertices. For different points in time the wavefronts are shown in gray.

The straight skeleton S(P) of a simple polygon P was introduced by Aichholzer et al. [1] and is defined by considering the propagation of a so-called wavefront. Each edge of P emits a wavefront-edge moving at unit speed towards the polygon's interior in a self-parallel manner. During this propagation process, the topology of the wavefront changes due to self-interaction: (i) In an *edge event* an edge of the wavefront shrinks to zero length and thus vanishes. (ii) A *split event* happens when a vertex of the wavefront moves into the interior of a non-incident wavefront edge. (iii) For input that is not in general position even more complex interactions such as *vertex-events* or *multi-split-events* are possible [2], [3]. The straight skeleton is the union of the traces of wavefront vertices over the entire time of the wavefront propagation, see Fig. 1.

The weighted version of the straight skeleton, where edges no longer move at unit speed, was first mentioned by Eppstein and Erickson [2] and studied in detail by Biedl et al. [4], [5]. Several algorithms are known for constructing unweighted straight skeletons, such as those by Aichholzer et al. [1], Eppstein and Erickson [2], Cheng and Vigneron [6], Huber and Held [3], or Vigneron and Yan [7].

**Fig. 2:**
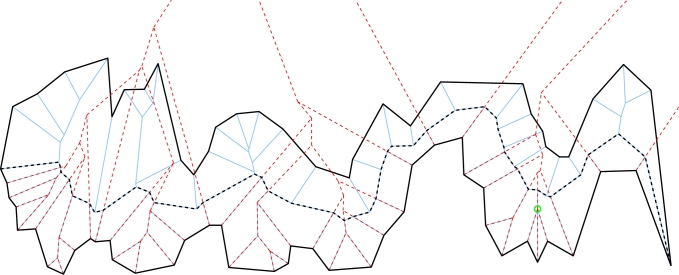
The straight skeleton S(P) of a strictly monotone input polygon P results from the merge of the straight skeletons $S(C_{u})$ and $S(C_{b})$ of the top and bottom chain – $S(C_{b})$ is shown dotted. The merge chain is shown in bold and dotted.

Das et al. [8] suggested an algorithm for constructing the (unweighted) straight skeleton of monotone polygons, which they claim runs in O(nlog⁡n) time, where *n* denotes the number of vertices of the polygon. However, we have simple examples that show that their Lemmas 5, 6, and 7 do not hold for all valid inputs. In particular, their approach hinges upon the assumption that no event introduces a new reflex vertex during the wavefront propagation process, which is clearly incorrect for general input. (See the node marked in green in Fig. 2, on the right-hand side of the lower chain.) Note that a perturbation of the input in order to avoid such a vertex event, as suggested by Das et al. [8], cannot be applied as the straight skeleton changes discontinuously [2].

Our algorithm can compute the positively weighted straight skeleton of a monotone polygon in O(nlog⁡n) time and O(n) space, which constitutes a significant improvement over the $O(n^{17/11+\epsilon })$ worst-case time and space complexity of the currently best algorithm for arbitrary simple polygons by Eppstein and Erickson [2]. (The algorithm by Vigneron and Yan [7] achieves an expected $O(n^{4/3}\log n)$ time complexity but is only applicable if no multi-split events occur.) Furthermore, our algorithm does not require complex data structures and is easy to implement.

## 2 Strictly monotone polygonal chains

Let C be a polygonal chain strictly monotone with respect to the *x*-axis. We construct $C^{\prime }$ as a chain consisting of the same line segments as C except that we extend the first and the last segment to rays to the west (x→−∞) and the east (x→+∞), respectively. Thus, $C^{\prime }$ partitions the plane into an upper and lower portion. In the following we continue to call such an unbounded chain a polygonal chain.

We start by considering the unweighted wavefront propagation of $C^{\prime }$ where all edges of $C^{\prime }$ emanate a self-parallel wavefront edge towards y→−∞, which we call the south. (We discuss extensions to positive weights and non-strict monotonicity later on.) We denote the wavefront at time *t* by $W_{C}(t)$. For the initial wavefront, at t=0, the chains $W_{C}(t)$ and $C^{\prime }$ are geometrically identical. Again, the first and last segments are rays to infinity. For small values of *t* and prior to the first event, the edges of $W_{C}(t)$ are in one-to-one correspondence to the edges of $C^{\prime }$ and occur in the same order.

We denote by $A_{C}(t)={⋃}_{0\leq t^{\prime }\leq t}W_{C}(t^{\prime })$ the area swept by the wavefront until time *t*. The *roof model* T(C) is a three-dimensional structure: We assign to every point of $A_{C}(\infty )$ the time when it was swept by the wavefront. The resulting structure $T(C)={⋃}_{0\leq t}(W_{C}(t)\times \{t\})$ is a terrain; it helps us in studying the wavefront over its entire propagation period.

Lemma 1 *The wavefront* $W_{C}(t)$ *of a strictly x-monotone chain* C *is a strictly x-monotone chain for all times t. Furthermore, every change in the topology of the wavefront is witnessed by an edge collapsing to zero length.*

Proof We will show this claim by induction: The monotonicity of $W_{C}(t)$ is unaffected by the southwards propagation at least as long as its topology does not change. Since the initial wavefront is identical to $C^{\prime }$, which is strictly *x*-monotone, it follows that $W_{C}(t)$ is also strictly *x*-monotone for sufficiently small *t* such that no event has happened yet.

The topology of $W_{C}(t)$ can only change as a result of the wavefront interacting with itself, when vertices of $W_{C}(t)$ become incident to other elements of $W_{C}(t)$ which they were previously not incident to. Assume that at a certain point in the propagation process a vertex *v* becomes incident to either a different vertex $v^{\prime }$ or to an edge *e* that it was not previously incident to. If prior to the change the wavefront was a strictly monotone polygonal chain, then it follows that the only way that *v* could have become incident to $v^{\prime }$ or *e* is for all segments between *v* and $v^{\prime }$ or *e* to have shrunk to zero length.

Segments that shrink to zero length in an event are removed from the wavefront. Removing one or more such collapsed segments of a strictly *x*-monotone chain and then connecting the remaining pieces together yields a strictly *x*-monotone chain again.

Therefore, each event is witnessed by an edge collapse and it transforms one strictly *x*-monotone wavefront into another strictly *x*-monotone wavefront, resulting in $W_{C}(t)$ being strictly *x*-monotone for all times *t*.  □

Note that we say that each change is witnessed by an *edge collapse* instead of an *edge event*. We do this in order to also include non-elementary changes of the wavefront, such as vertex events.

Theorem 2 *The straight skeleton* S(C) *of a strictly monotone polygonal chain* C *can be computed in time* O(nlog⁡n) *, where n is the number of edges of* C *.*

Proof We maintain a priority queue to keep track of the edge collapses, which witness all topological changes. The total number of propagating wavefront edges initially is *n*. We can compute the initial collapse times of all these edges in O(n) time and fill a priority queue in total O(nlog⁡n) time.

We keep fetching the next edge collapse from the priority queue. We have to re-compute the collapse times of the two incident edges and adapt their entries in the priority queue in O(log⁡n) time. Collapse times of other edges are not affected by such a change. Note that the number of wavefront edges decreases with each event because no split events occur.

Since all topological changes of the wavefront are witnessed by such edge collapses (Lemma 1), no event is missed. Therefore, we can construct the straight skeleton S(C) in total O(nlog⁡n) time.  □

Lemma 3 *Each wavefront edge of* $W_{C}(t)$ *has area already swept by the wavefront to its immediate north and unswept area to its south.*

Proof The part of the plane that has already been swept, $A_{C}(t)$, lies between $C^{\prime }$ and $W_{C}(t)$. Since $W_{C}(t)$ is strictly *x*-monotone, each element of $W_{C}(t)$ has swept area to its north and unswept area to its south.  □

Lemma 4 *Let* T(C) *be the roof of a strictly monotone chain* C *that emanated a wavefront southwards. Then, on any point on the surface of* T(C) *, the elevation of the roof increases when moving due south.*

Proof This holds for each individual face of T(C) because of Lemma 3. Since the roof is continuous, it also holds when moving between faces.  □

## 3 Strictly monotone polygons

To compute the unweighted straight skeleton of a strictly monotone polygon P, which we assume to be monotone with respect to the *x*-axis, we split P into two chains, the northern or top (upper) chain $C_{u}$ and the southern or bottom chain $C_{b}$. The common western and eastern vertices are denoted by $v_{w}$ and $v_{e}$, respectively. Both chains of P emanate their wavefronts inwards, that is, $C_{u}$ emanates its wavefront southwards, $C_{b}$ northwards. We compute the straight skeleton and roof for both chains independently and denote these by $S(C_{u})$, $S(C_{b})$, $T(C_{u})$, $T(C_{b})$.

Definition 1 Let M be a polygonal chain in $R^{3}$. We say M is *strictly* 3*D-monotone* with respect to the *x*-axis if every plane parallel to the *yz*-plane intersects M in at most one point.

Lemma 5 *Let*M*be a polygonal chain in*$R^{3}$*that is strictly* 3*D-monotone with respect to the x-axis. Then projecting* M *onto the xy-plane yields a polygonal chain* $M^{\prime }$ *that is strictly x-monotone.*

Proof Let *h* be an arbitrary line in the *xy*-plane orthogonal to the *x*-axis (i.e., parallel to the *y*-axis). To establish that $M^{\prime }$ is *x*-monotone, we need to show that *h* intersects $M^{\prime }$ in at most one point.

Let *H* be a plane parallel to the *yz*-plane such that *h* lies within *H*. By assumption, *H* intersects M in at most one point. Thus, the projection of M onto the *xy*-plane intersects *h* in at most one point.  □

Lemma 6 *The two roofs*$T(C_{u})$*and*$T(C_{b})$*intersect in a polygonal chain*M*in*$R^{3}$*that is strictly* 3*D-monotone with respect to the x-axis. This* merge chain *starts in* $v_{w}$ *and ends in* $v_{e}$*.*

Proof Monotonicity of the merge chain M follows from Lemma 4: Let *Π* be an arbitrary plane parallel to the *yz*-plane that intersects $W_{C_{b}}(0)$ in $p_{b}$ and $W_{C_{u}}(0)$ in $p_{u}$. If *Π* intersects the polygon P then $p_{b}$ is the southern intersection and $p_{u}$ the northern intersection of *Π* and P.

The intersection of *Π* with $T(C_{b})$ is a piecewise linear terrain function in *Π* that starts at point $p_{b}$, where z=0, and strictly monotonically increases its *z*-coordinate as the *y*-coordinate increases towards y→+∞. Similarly, the intersection of *Π* with $T(C_{u})$ is a strictly monotonically decreasing function starting at y→−∞ and positively infinite *z*-coordinate and ending at point $p_{u}$ where z=0.

Two such functions coincide in exactly one point if $p_{u}$ is north of $p_{b}$, as is the case if *Π* intersects the polygon. If the intersection of *Π* and P is empty, then $p_{b}$ is north of $p_{u}$ and the two terrain functions do not intersect at all. Therefore, M is strictly 3*D*-monotone with respect to the *x*-axis.

Vertices $v_{w}$ and $v_{e}$ are the start and end points of the chain because they are the common vertices of both chains. Since both roofs consist of planar faces, the intersection consists of line segments.  □

In the merge step, we construct a new polyhedron T by stitching together the faces of $T(C_{u})$ between $C_{u}$ and M and the faces of $T(C_{b})$ between $C_{b}$ and M. Note that this polyhedron is a terrain above the interior of P and its intersection with the *xy*-plane is equal to P. Furthermore, T is piecewise-linear and continuous and each face is incident to one edge of P. It remains to show that this roof T is equivalent to the straight-skeleton induced roof T(P) of P.

Lemma 7 *All edges introduced by our merge step, i.e., the edges of* M *, are ridges in* T *, not valleys.*

Proof Each edge *e* of M is incident to one face $f_{u}$ of the northern roof on its north side, and incident to one face $f_{b}$ of the southern roof to its south. By Lemma 4, $f_{u}$ is sloping downwards towards north, and $f_{b}$ is sloping downwards towards south. Thus, *e* is a ridge.  □

Theorem 8 *Let* T *be the roof constructed by merging* $T(C_{u})$ *and* $T(C_{b})$ *as described. Then* T *is the roof induced by* S(P) *.*

Proof In the following we will only consider the roof above P. Note that T is the unique lower envelope of $T(C_{u})$ and $T(C_{b})$. Let T(P) denote the roof induced by S(P). We need to show that T(P) also is the lower envelope of $T(C_{u})$ and $T(C_{b})$.

As S(P) is a tree, there is a unique path $M_{S}^{\prime }$ between $v_{w}$ and $v_{e}$. This is exactly the path that separates the union of faces incident to $C_{u}$ and the union of faces incident to $C_{b}$. Hence, $M_{S}^{\prime }$ comprises exactly the straight-skeleton arcs that have faces of different chains on either side. From that it follows that $M_{S}^{\prime }$ is *x*-monotone.

We build vertical slabs above the edges of $M_{S}^{\prime }$, resulting in the intersection $M_{S}$ with $T(C_{b})$. All straight-skeleton nodes of S(P) south of $M_{S}^{\prime }$ originate from topological changes within $C_{b}$. Hence, their lifted counterparts in T(P) coincide with vertices of $T(C_{b})$. Likewise, every vertex in $T(C_{b})$ south of $M_{S}$ has its counterpart as a node of S(P) south of $M_{S}^{\prime }$. In other words, $T(C_{b})$ and T(P) coincide south of $M_{S}^{\prime }$. With the same argument, $T(C_{u})$ and T(P) coincide north of $M_{S}^{\prime }$. In particular, $M_{S}=T(C_{b})\cap T(C_{u})$.

Finally, we observe that every path on T(P) from $M_{S}$ strictly to the north or the south is descending. On the other hand, a path on $T(C_{u})$ to the south is ascending. The same is true for paths on $T(C_{b})$ to the north. Hence, T(P) is indeed the lower envelope of $T(C_{u})$ and $T(C_{b})$.  □

Fig. 2 illustrates the merge by showing a polygon P and its straight skeleton S(P) as well as the straight skeleton of the bottom chain $S(C_{b})$.

Corollary 9 *Projecting* T *onto the xy-plane yields the straight skeleton of the input polygon* P *.*

## 4 Computing the straight skeleton

##### Computing the merge chain

We construct the intersection M of the northern and southern roofs. The roofs and intersections that appear in this problem have special properties, and we can, thus, find M in O(nlog⁡n) time.

Since in the end we are only interested in the projection of M onto the *xy*-plane, we only construct this projection $M^{\prime }$ in 2*D*. The three-dimensional chain M can be extracted from $M^{\prime }$ by raising each vertex by its orthogonal distance to the edges defining its incident faces.

Let $C_{u}$ consist of the edges $e_{u,1},e_{u,2},e_{u,3},\ldots ,e_{u,N_{u}}$, in order from west to east, and $C_{b}$ of the edges $e_{b,1},e_{b,2},e_{b,3},\ldots ,e_{b,N_{b}}$. Furthermore, let $M^{\prime }$ consist of vertices and edges $v_{0}=v_{w},m_{1},v_{1},m_{2},v_{2},m_{3},v_{3},\ldots ,v_{N_{m}-1},m_{N_{m}},v_{N_{m}}=v_{e}$.

We construct $M^{\prime }$ incrementally, starting at $v_{w}$ and adding line segments $m_{i}$ until we reach $v_{e}$. At each step in the process we keep track of the faces of $S(C_{u})$ and $S(C_{b})$ that $m_{i}$ lies in. We denote these faces by $f_{u,i}$ and $f_{b,i}$. Note that $m_{i}$ lies on the bisector of the input edges defining $f_{u,i}$ and $f_{b,i}$ since $M^{\prime }$ traces the intersection of the northern and southern roofs.

The initial merge segment $m_{1}$ starts at $v_{0}=v_{w}$. The northern and southern faces are $f(e_{u,1})$ and $f(e_{b,1})$, where f(e) denotes the face incident to edge *e* in the corresponding straight skeleton. The supporting line of $m_{1}$ will intersect arcs of $S(C_{u})$ and $S(C_{b})$ in loci other than $v_{0}$. Let $v_{1}$ be the intersection east of $v_{0}$ that minimizes the length of the edge $m_{1}=\bar{v_{0}v_{1}}$.

Assume $v_{1}$ was the intersection of the supporting line of $m_{1}$ with an arc *a* of the northern straight skeleton $S(C_{u})$. We set the northern face $f_{u,2}$ to be the other face incident to *a*. The southern face does not change and thus $f_{b,2}=f_{b,1}$. Should the intersection have occurred for an arc of the southern face, we set $f_{u,2}$ and $f_{b,2}$ accordingly. The next segment, $m_{2}$, then lies on the bisector of the input edges defining $f_{u,2}$ and $f_{b,2}$. This segment starts in $v_{1}$ and we determine the next vertex $v_{3}$ in turn.

Since the segments of $m_{i}$ always lie between the northern and southern chain, and since $M^{\prime }$ is monotone, this process will, eventually, end up in $v_{e}$ and we will have completed the merge chain.

##### Complexity considerations

In order to quickly find the next intersection of $m_{i}$ with an arc of either $S(C_{u})$ and $S(C_{b})$ we compute trapezoidations of $S(C_{u})$ and $S(C_{b})$ by adding vertical line segments as required.

In the incremental step, when we are looking for $v_{i}$, i.e., the eastern vertex of $m_{i}$, we can use the trapezoidations of both $f_{u,i}$ and $f_{b,i}$ to find $v_{i}$: We sweep through the cells from west to east until we find one where the supporting line of $m_{i}$ intersects an arc on a cell's boundary, thus finding $v_{i}$. During that switch we enter a new face in either the northern or the southern straight skeleton and we start anew. When searching for $v_{i+1}$ in this next step, we no longer need to consider the cells that we already visited.

At every stage we intersect $m_{i}$ with the boundary of the currently active northern and southern cells to either find $v_{i}$ or to move forward in one of the trapezoidations. We can charge each such set of intersection tests to the one cell we move out of. The number of total trapezoidation cells is linear in the input size, and, therefore, given a trapezoidation, the complete merge step can be achieved in time linear in the input size.

A plane sweep allows to compute the trapezoidation of a planar straight-line graph such as $S(C_{u})$ in O(nlog⁡n) time, where *n* is the size of the input graph. Since the size of the straight skeleton is linear in its input, the total cost of creating both trapezoidations therefore also is in O(nlog⁡n).

By Theorem 2, the northern and southern straight skeletons can be found in O(nlog⁡n) time, and they can also be merged in O(nlog⁡n) time. Therefore, the unweighted straight skeleton of a strictly monotone polygon can be computed in O(nlog⁡n) time. Obviously, all data structures require space linear in the input size.

## 5 Positively weighted straight skeleton

We now consider a wavefront propagation where not all edges move at the same speed. Recently Biedl et al. [4] showed that many of the seemingly obvious properties of straight skeletons no longer hold when weights are not unit weights. Therefore, diligent consideration is required when extending existing proofs to weighted straight skeletons.

##### Monotone chains

None of the proofs of any statement leading up to and including Theorem 2 used the fact that wavefront edges move at unit speed or any properties which depend on unit speeds.

Thus, Theorem 2 still holds and the positively weighted straight skeleton and roof of a polygonal chain can be computed in O(nlog⁡n) time.

Since we consider only positive weights, the wavefront of a strictly monotone chain still propagates southwards everywhere. Thus, Lemma 3 and Lemma 4 still hold, and the straight-skeleton induced roof is still a terrain.

##### Monotone polygons

For strictly monotone polygons the positively weighted straight skeleton can also be constructed in the way described in Sections 3 and 4. Since Lemma 4 still holds, the intersection of the northern and southern roofs again produces a 3*D*-*x*-monotone polygonal chain that consists of ridges only. Therefore, Lemma 6, Lemma 7 also hold for positively weighted input. Biedl et al. [4] showed that for simple polygons and positive weights the straight skeleton is still a tree. Thus, Theorem 8 still applies and therefore also Corollary 9. Hence, our algorithm is also correct for positively weighted straight skeletons.

## 6 Discussion

We presented an algorithm that computes the positively weighted straight skeleton of a strictly monotone polygon in O(nlog⁡n) time and linear space. The restriction to strict monotonicity makes several proofs easier and more readable but can be waived. The obvious problem when dealing with a polygon which, after suitable rotation, is monotone but not strictly monotone relative to the *x*-axis is that moving due northwards or southwards in $T(C_{u})$ and $T(C_{b})$ does no longer guarantee that the elevation increases or decreases, respectively. Furthermore, the merge chain $M_{S}^{\prime }$ need no longer be strictly monotonous.

We refrain from adding clumsy new proofs to extend our lemmas to this special case. Rather, we content ourselves with noting that suitable subdivisions of the top and bottom chains (in order to isolate edges parallel to the *y*-axis within individual chains) followed by suitable (small) rotations allow to obtain the properties claimed by our lemmas locally for the individual pieces of the chains, their roofs, and the final merge chain $M_{S}^{\prime }$. Thus, our algorithm can be extended to polygons that are monotone but not strictly monotone, without sacrificing its performance.

An extension of our approach to negative weights seems much more demanding since several important properties are lost. For instance, the roof of a monotone polygonal chain need not be a terrain once negative weights are allowed. For our approach to still work we would need to establish that the northern and southern roofs nevertheless still only intersect in a single, unique merge chain. Note that this merge chain need no longer be monotone, might include vertices at infinity, and its projection onto the *xy*-plane might self-intersect. The straight skeleton of a monotone polygon might contain cycles, and even for a convex polygon it may be self-intersecting [4].
